# Delivering high-resolution landmarks using inkjet micropatterning for spatial monitoring of leaf expansion

**DOI:** 10.1186/1746-4811-7-1

**Published:** 2011-01-25

**Authors:** Lisheng Wang, Simon T Beyer, Quentin CB Cronk, Konrad Walus

**Affiliations:** 1 Department of Electrical and Computer Engineering, The University of British Columbia, 2332 Main Mall, V6T1Z4, Vancouver, BC, Canada; 2 Department of Botany and Faculty of Land and Food Systems, The University of British Columbia, 6270 University Blvd., V6T1Z4, Vancouver, BC, Canada

## Abstract

**Background:**

Inkjet micropatterning is a versatile deposition technique with broad applications in numerous fields. However, its application in plant science is largely unexplored. Leaf expansion is one of the most important parameters in the field of plant science and many methods have been developed to examine differential expansion rates of different parts of the leaf lamina. Among them, methods based on the tracking of natural landmarks through digital imaging require a complicated setup in which the leaf must remain fixed and under tension. Furthermore, the resolution is limited to that of the natural landmarks, which are often difficult to find, particularly in young leaves. To study the fine scale expansion dynamics of the leaf lamina using artificial landmarks it is necessary to place small, noninvasive marks on a leaf surface and then recover the location of those marks after a period of time.

**Results:**

To monitor leaf expansion in two dimensions, at very fine scales, we used a custom designed inkjet micropatterning system to print a grid composed of c. 0.19 mm^2 ^cells on small developing leaves of ivy (*Hedera helix*) using 40 μm dots at a spacing of c. 91 μm. The leaves in different growing stages were imaged under magnification to extract the coordinates of the marks which were then used in subsequent computer-assisted leaf expansion analyses. As an example we obtained quantified global and local expansion information and created expansion maps over the entire leaf surface. The results reveal a striking pattern of fine-scale expansion differences over short periods of time. In these experiments, the base of the leaf is a "cold spot" for expansion, while the leaf sinuses are "hot spots" for expansion. We have also measured a strong shading effect on leaf expansion. We discuss the features required to build an inkjet printing apparatus optimized for use in plant science, which will further maximize the range of tissues that can be printed at these scales.

**Conclusions:**

To apply inkjet micropatterning to plant studies, we have successfully delivered landmarks on ivy leaf surfaces and achieved high-resolution, two-dimensional monitoring of leaf expansion at different growing stages. The measurement is capable of reliably identifying the fine scale changes during plant growth. As well as delivering landmarks, this technology may be used to deliver microscale targeted biological components such as growth hormones, and possibly be used to pattern sensors directly on the leaves.

## Background

Inkjet printing is a technique involving ejecting tiny liquid droplets in a non-contact manner onto target object surfaces to form any desired patterns in high resolution. Historically, it has been used almost exclusively for document and image printing, finding considerable success in the consumer electronics market. More recently the potential of this technology has been explored to a greater degree, especially in developing printable electronics. Popular applications include polymer solar cells, thin film transistors, sensors and organic light emitting diode arrays [1]. If functional materials and patterns are printed directly on plant bodies, various plant behaviours can be accurately investigated in exquisite detail which raises the possibility of "sentinel plants" reporting on growth conditions (desiccation, gas exchange, photon flux density etc.). The non-contact nature of inkjet printing makes it a suitable tool for depositing materials on plant surfaces without causing physical damage. Therefore, we consider the application of inkjet printing to plant studies is not only of great significance but also very feasible. However, this area has scarcely been explored until now.

Among various topics in botany, precise understanding of the growth of plant organs is important not only in the study of morphogenesis but also in the applied plant sciences, as the expansion of the leaf lamina is an important factor contributing to plant yield. Change in total leaf area is easy to measure as the leaf outline is easily captured. However, differential expansion of different parts of the leaf is more difficult to study as natural landmarks within the leaf are difficult to find.

The pattern of veins can provide landmarks, particularly if the venation is finely reticulate, but at early stages the veins are poorly formed and the pattern changes rapidly. Nevertheless, for later stages of leaf development the question of whether various parts of the lamina expand at the same or at different rates throughout development has been answered using the expansion of small areas delineated by veins[2]. However, this method cannot collect measurements with a high spatial resolution.

In a more recent study by Walter *et al.*[3], leaf venation was tracked using advanced digital image sequence processing, resulting in a method with very high temporal resolution. However, the spatial resolution of this method and the requirement that the leaves remain mechanically fixed to the apparatus during growth makes it more suitable for large leaves.

Finer scale measurements can be achieved with clonal analysis in which individual cell lineages are followed using phenotypic markers [4-7] but this depends on the availability of suitable biological material. It has successfully been used to study leaf and petal expansion using visible changes mediated by, for instance, transposon activity.

Another method involves placing artificial landmarks on the plant organ. Avery [8], in a pioneering study of leaf expansion, marked a grid on a developing tobacco leaf using India ink. This technique is only suitable for relatively large leaves, typically tobacco leaves c. 6 cm long, and using a fairly coarse initial landmark spacing (c. 5 mm). Numerous subsequent authors have used the same technique [9-11]. A modern variant of this procedure is to use graphite particles scattered randomly [12]. This technique can reach much finer scales but requires careful image analysis to reconstruct the landmarks as they move apart during expansion.

To address some of these problems of artificial landmarks, Hamamoto, Kimura & Yamaguchi [13] used non-contact inkjet printing to print lines of small dots (0.1-0.2 mm in diameter) on the surfaces of plant organs. However, the authors only studied the elongation of the organs along one direction. The purpose of this paper is to extend the inkjet micropatterning method, printing at much finer scales and in two dimensions, using 40 μm dots, in order to study the expansion of very young leaves. We used ivy (*Hedera helix *cv) as the experimental material. The complex outline of its palmate-shaped leaves allowed us to study the role of differential expansion in the morphogenesis of such complex shapes.

## Methods

### Plant material and growth

**Figure 1:**
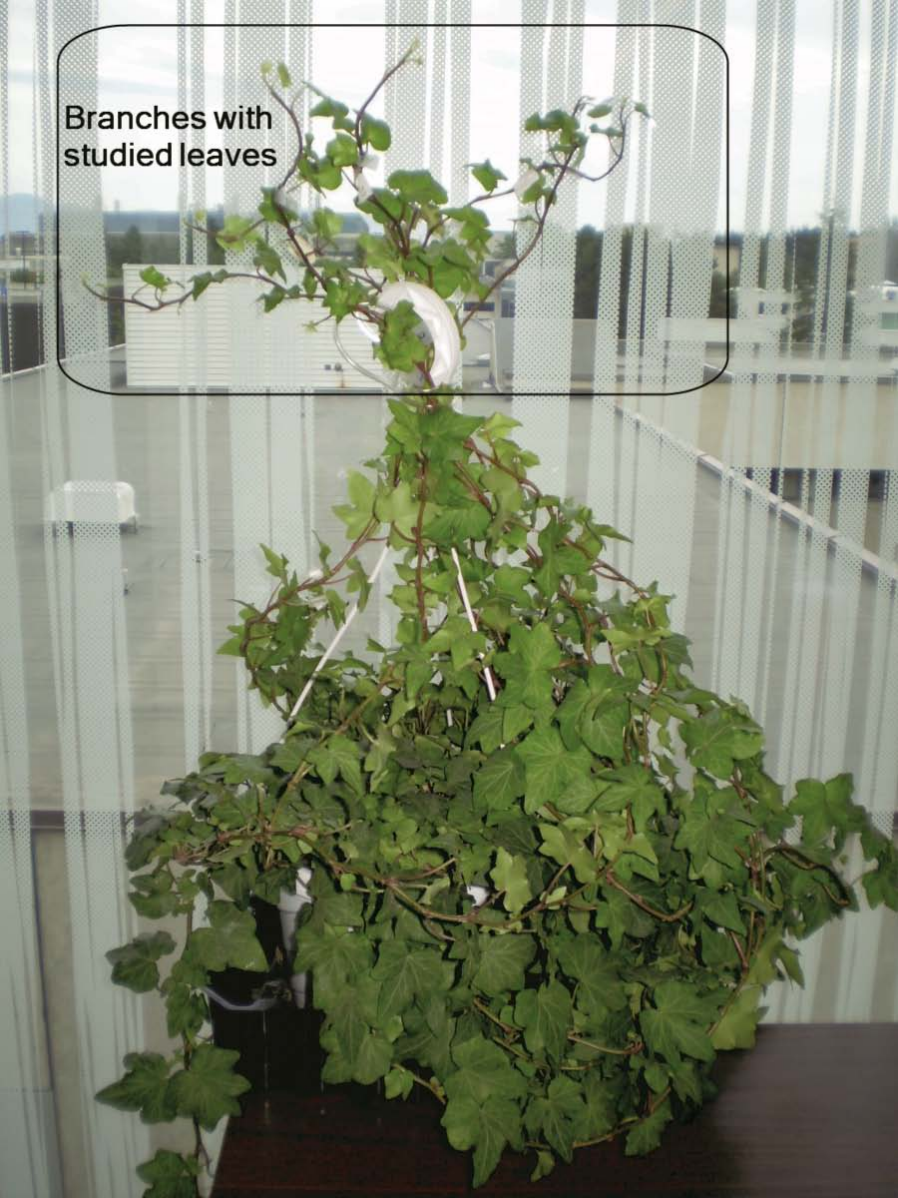
**Photograph of studied ivy plant**. The branches with studied leaves were supported at the top of the plant and separated from others.

The plant used in the experiments was English ivy (*Hedera helix *cv) shown in Figure 1. It was grown in a 20 cm-diameter pot positioned with side illumination from a window and kept at a relatively constant temperature of c. 25 °C. The plant was able to receive direct sunlight from the southeast for approximately five hours per day throughout the experimental period (summer). It was watered two to three times per week and supplied with nutrients using houseplant nutrient spikes (Miracle-Gro^®^, The Scotts Co.) once before the experiment to ensure that leaf growth was unaffected by nutrient or water deficits.

The ten studied leaves were selected from ten primary branches of the plant. Each leaf was 17-20 mm long at Day 0 of the experiment, corresponding to a leaf plastochron index (LPI) of c. 0.8-0.9 [2] assuming that the LPI of a 10 mm long leaf is set at zero by convention. The leaves were measured at the 5th, 10th and 15th day, corresponding to a growth interval of c. 0.3, 0.6 and 0.9 plastochrons respectively (where one plastochron is the interval between the production of successive leaves on a shoot). The branches bearing the leaves under study were supported at the top of the plant and separated from others to make sure they received ample natural light distributed as evenly as possible (Figure 1).

### Pattern printing on leaf surfaces

**Figure 2:**
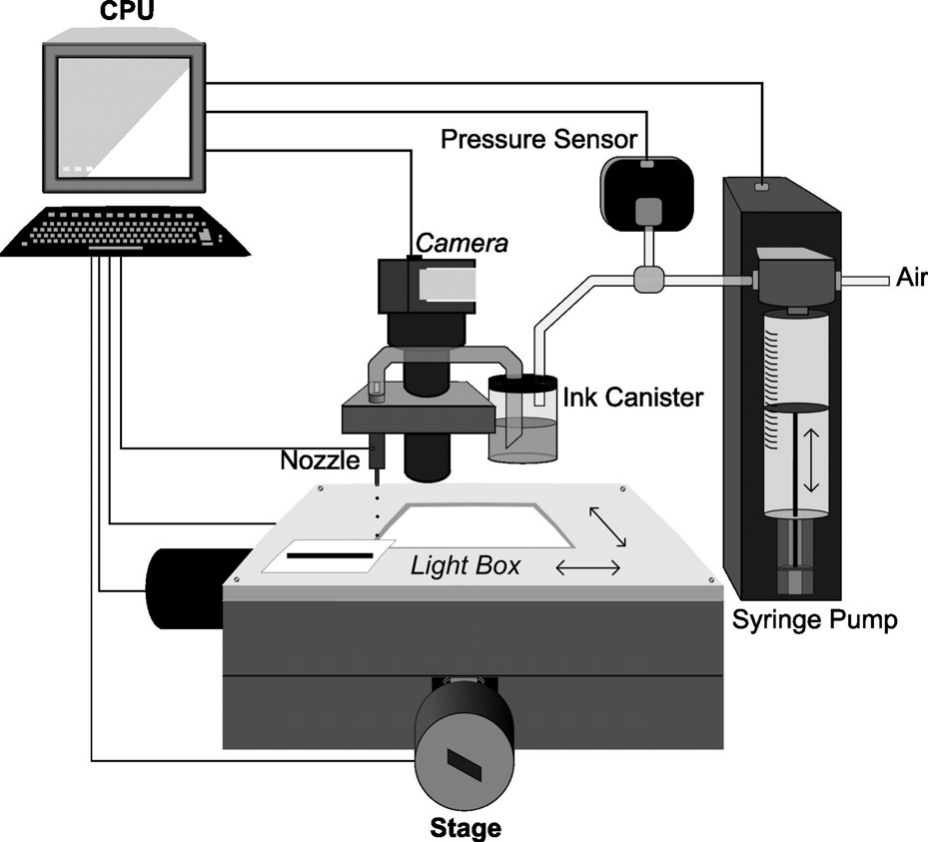
**Schematic diagram of the inkjet printing/imaging system**. The inkjet printing system includes a nozzle, pressure sensor, ink canister, syringe pump, CPU and stage, and the imaging system includes a camera, light box, CPU and stage.

The inkjet printing was performed using a custom-built drop-on-demand micropatterning system. Figure 2 shows the schematic diagram of this system. We used a 40 μm orifice diameter piezoelectrically-driven nozzle (MJ-AB-63-40; MicroFab Technologies, Inc.). When actuated with a voltage pulse, the nozzle was able to eject droplets of ink on the order of picoliters per drop, forming individual dots on the leaves. The voltage level used for actuation was 29 V with a 92 μs wide sinusoidal pulse. A syringe pump (PSD/3 5175-01; Hamilton Inc.) combined with an inline pressure sensor (PX139-001D4V; OMEGA, Inc.) was used to control the air pressure in the ink canister in order to maintain consistent droplet formation as ink was consumed.

To reduce nozzle vibration, the nozzle was kept stationary while the deposited pattern was formed through the movement of a two-dimensional stage. Stage movement with resolution of 2.5 μm was accomplished through the use of two stepper motors (23D - 6306C; American Precision Industries). Each leaf, along with the branch, was fastened onto the stage before printing by folding a piece of weakly adhesive tape, applying it to the back of the leaf and gently pressing to flatten it. The ink used was violet food colouring (Scott Bathgate Ltd.) which provided distinguishable contrast to the green leaves.

All components of the inkjet printer were controlled with a PC hosting custom C++ based software. Macro based commands were used to determine the patterns to be printed on the leaves. The pattern used was a square grid consisting of 56 horizontal lines and 56 vertical lines; sufficient to cover the entire surface of each leaf. Each line was composed of individual dots with a centre-to-centre distance of 87 μm. Line spacing in both the vertical and horizontal directions was 435 μm.

### Pattern recording

The recording of printed patterns was carried out on the same 2D stage (Figure 2). A USB CMOS camera (EO-1312M; Edmund Optics, Inc.) with a 0.42× zoom lens (HRP042-CMT; Diagnostic Instruments, Inc.) was mounted beside the nozzle and connected to a computer. Uc480Viewer (Thorlabs, Inc.) was used to capture digital images. A home-built LED light box was attached to the top of the stage to provide backlighting during imaging. Each leaf was placed on the window of the light box and gently covered with a glass slide to fix and flatten its surface. The stage was stepped in a grid pattern to produce a total of 9-16 photographs (depending on the size of the leaf) of different regions of the leaf. A panoramic image was then created by stitching those photographs together using Windows Live Photo Gallery 2009 (Microsoft Co.).

### Pattern extraction and computer-assisted analysis

The method and algorithm to acquire quantitative and visualized expansion information of one leaf is composed of the following steps:

1. Manually rotate the leaf image with the initial printed pattern until the printed lines are approximately parallel to the horizontal and vertical axes of the screen.

2. Open the rotated image file with ImageJ 1.43j (National Institute of Health of United States; NIH). Manually select the intersecting points of the crossing lines, as well as the intersecting points of the printed lines and the leaf margin, using ImageJ to store the coordinates. If two or more points are very close to each other, select only one of them. Save the index numbers and coordinates of all selected points in a text file and those of margin points again in a separate text file.

3. The data analysis program, written using Matlab R2008a (The Mathworks, Inc.), is used to import the text files. The program takes each point from the text file and locates its neighbouring points by searching for the nearest one along the ascending *x *and *y *axis respectively as well as any point in its first quadrant to which the distance is no more than 650 μm (a little longer than times line spacing). Thus, the program will find three neighbours for points within the leaf and 0-4 neighbours for points along the leaf margin. The program discards those points with no more than one neighbour as well as those without neighbours along both the ascending ¬*x *and *y *axes. It is worth mentioning that the high accuracy of the inkjet printing process makes automatic selection of neighbouring points a feasible possibility.

4. Each point and its neighbouring points specify a small square or triangular cell. Here, and for the remainder of this paper, a cell is defined as the region between printed lines rather than in the biological sense. The program records the index numbers of those points and calculates the area of the cell (*A*_0_) using the *polyarea *function. It also discards those cells which are outside of the leaf surface (specified by the recorded margin points) using the *inpolygon *function. The surface area of the entire leaf can be calculated either based on the polygon specified by the margin points or as the summation of the areas of all cells. Further study shows the difference between these two values of all studied leaves is less than 3%, which is negligible.

5. For distorted patterns at Day *x *after leaf expansion, repeat Steps 1 and 2. Select the points in the exact same order as in Step 2. That is, each point is expected to have the same serial number in the data files before and after leaf expansion.

6. The program calculates the areas of expanded cells (*A*_*x*_) specified in Step 4 as well as total cell expansion (*E*_*x*_) and relative expansion rates (*GR*_*x-y*_), defined as:

(1) $$E_{x}=\frac{A_{x}}{A_{0}}$$

(2) $$GR_{x-y}=\frac{A_{y}-A_{x}}{A_{x}\left(y-x\right)}$$

7. Lastly the program creates a simple leaf expansion map using the *patch *function and a contour map using the *contourf *function.

Computer programs and scripts used in this study are available upon request.

## Results

### Pattern printing and data acquisition

A total of ten young actively growing ivy leaves were selected, which were of similar developmental age and size at the start of the experiments (Table 1). We used a custom inkjet printer for non-contact printing on six of the leaves (Leaves 1-6) using commercial food colouring as the printing material.

**Table 1:** Size information, expansion and relative expansion rate of leaves at different time periods.

Leaf No.	Initial size		Expansion (mm^2^/mm^2^)			Relative expansion rate (mm^2^/mm^2^/day)			Experimental details
	Length (mm)	Area (mm^2^)	Day 5	Day 10	Day 15	Day 0-5	Day 5-10	Day 10-15	
1	20	184	2.7	3.5	3.7	0.33	0.064	0.012	Patterned.
2	18	159	2.6	3.4	3.7	0.32	0.065	0.013	
3	20	214	1.7	1.8	1.9	0.13	0.019	0.007	
4	20	195	1.5	1.6	1.6	0.10	0.021	0.006	
5	17	169	1.3	1.4	1.4	0.05	0.019	0.008	Patterned; partly covered.
6	18	202	1.4	1.6	1.7	0.08	0.034	0.010	

7	19	176	1.9	2.2	2.3	0.17	0.038	0.009	Controls.
8	19	180	1.5	1.7	1.7	0.10	0.020	0.006	
9	20	193	2.3	2.7	2.8	0.25	0.040	0.007	
10	17	163	2.5	3.3	3.5	0.30	0.067	0.011	

**Figure 3:**
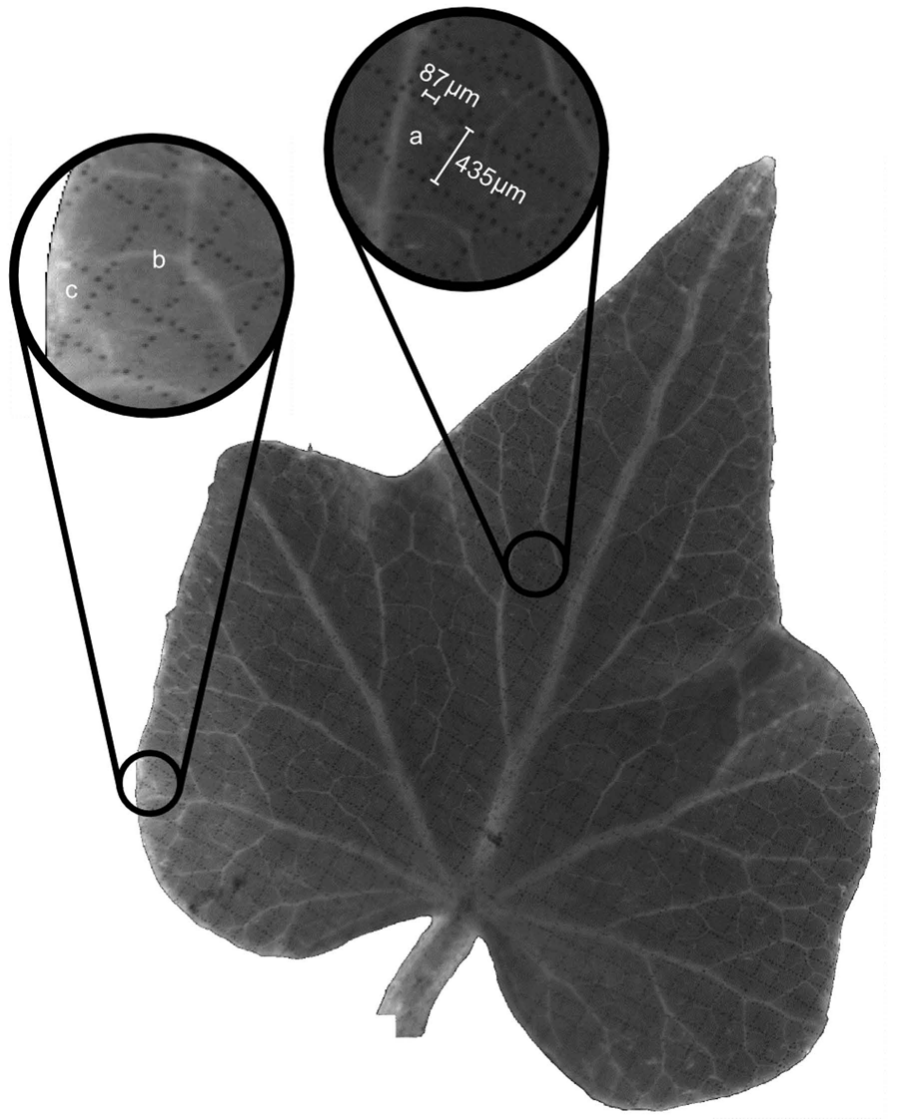
**One ivy leaf (Leaf 4) with printed grids on its surface**. Each grid line is composed of discontinuous dots. The entire surface is divided into 700 small cells. Regions a (square), b (deformed square) and c (triangle) represent different shapes of the cells.

As shown in Figure 3, each leaf is marked with a high-resolution square grid. Each grid line is patterned with discontinuous dots of c. 40 μm in diameter. The average distance between two adjacent dots is 91 μm and the average spacing between two parallel lines is 435 μm (Inset of Figure 3). The entire surface of each leaf is divided into a grid of 700-1000 small cells depending on the size of the leaf. Most cells are located on the planar portions of the leaves and are approximately square-shaped with an average area of 0.19 mm^2 ^(region 'a' in inset of Figure 3). Some cells are somewhat deformed because of the curved surface (Region 'b' in inset of Figure 3) and those near the margin may have other shapes, e.g. triangles, trapezoids or pentagons, etc. (Region 'c' in inset of Figure 3). Fortunately, the shape of the cells does not influence the quantitative expansion analysis since we are measuring their relative expansion.

To study the influence of the inkjet printing process on leaf viability, four additional leaves were selected as unprinted controls (Leaves 7-10).

**Figure 4:**
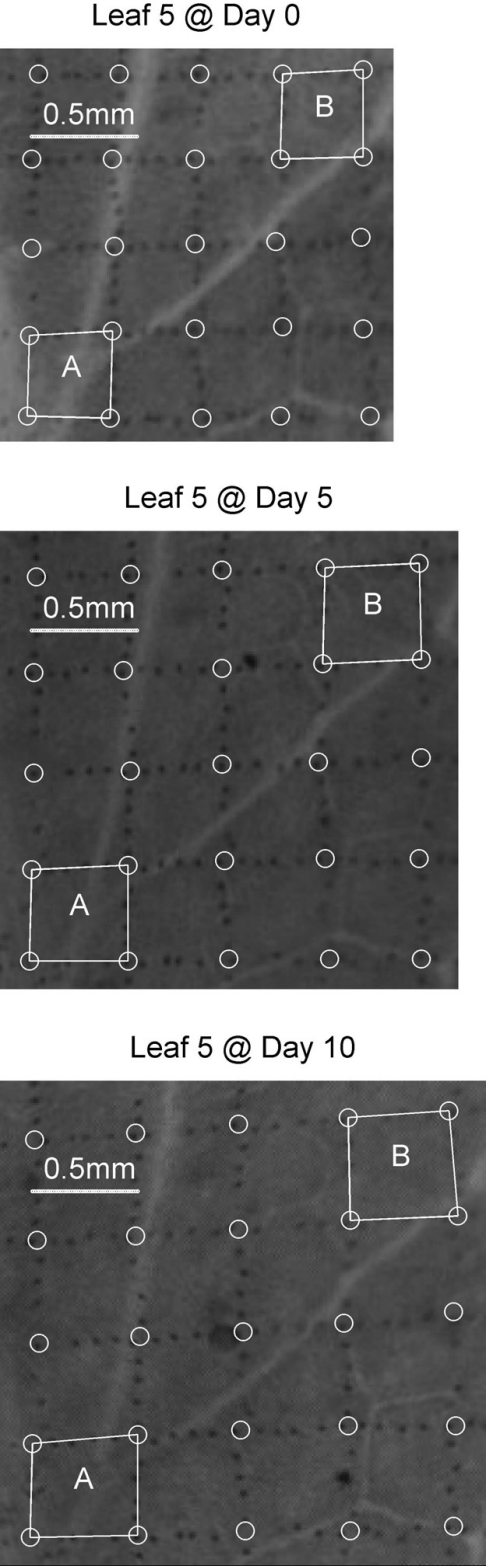
**A small region of Leaf 5 at different growing stages showing the change of printed grid**. 25 example points (white circles) and two marker-defined cells (Cell A and Cell B) were labelled. Each cell does not experience the same rate of expansion. Cell A is 1.37 times larger at Day 10 than at Day 0 while Cell B is 1.62 times larger.

The studied leaves were monitored three times at five day intervals throughout the experiment. In other experiments, more frequent measurements over longer intervals could certainly be available if necessary. The patterned leaves were non-destructively imaged and the patterns were extracted and digitized manually for analysis. The intersection points between crossing lines and between printed lines and leaf margins were marked and their coordinates recorded. Figure 4 shows 25 example points (white circles) and two marker-defined cells (Cell A and Cell B). Those recorded coordinate values from each measurement are fundamentals in our monitoring system, with which we are able to perform various analyses using different models of interest. The following leaf expansion quantification, global expansion mapping and contour plotting are three demonstrating examples.

### Quantifying leaf expansion

With the acquired coordinates, we are able to calculate the area of each marker-defined cell. By recording the displacement of each printed point and the expansion of each cell, we were able to track the local expansion patterns of each leaf.

As an example, Figure 4 shows the morphology of a small 16 cell region of Leaf 5 over a 10 day span. The initial area of this region was 3.08 mm^2^. At Day 5 and Day 10 the total area increased to 4.05 mm^2 ^(1.3 times) and 4.57 mm^2 ^(1.5 times) respectively. From the image it is clear that each cell does not experience same rate of expansion. For example, Cell A in Figure 4 is 1.37 times larger at Day 10 than at Day 0 while Cell B is 1.62 times larger. This difference demonstrates the highly local expansion differences on the leaf.

The non-uniformity of leaf expansion is also evident in the deformation of the printed grid. In Figure 4, the top two horizontal lines are parallel to the bottom horizontal line at Day 0, but by Day 10 they are clearly no longer parallel. We believe this is due to a faster expansion rate in the upper right region of the leaf.

We are also able to determine the overall expansion of one leaf by tracking the surface area increase of the entire leaf. The average expansion of the patterned leaves (Leaves 1-4) is 2.1 at Day 5, 2.5 at Day 10 and 2.6 at Day 15 while those of controls (Leaves 7-10) are 2.0 at Day 5, 2.5 at Day 10 and 2.6 at Day 15. The patterned leaves grew at a similar rate as the controls, suggesting that the inkjet printing process does not have any significant influence on the expansion of the leaves.

**Figure 5:**
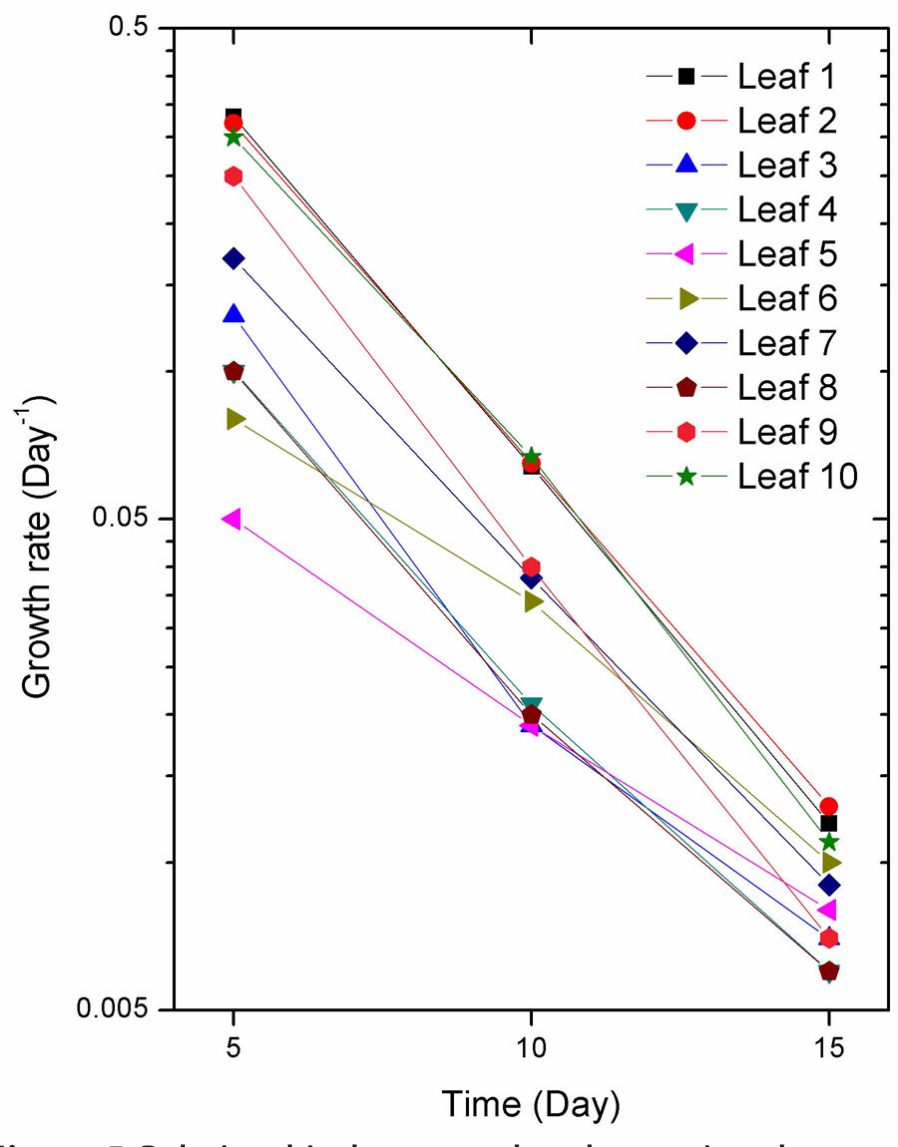
**Relationship between the observation date and expansion rate of ten studied leaves**. The relationship is approximately log-linear with an average correlation coefficient of 0.996 among the ten leaves studied.

The mean relative expansion rates at different time periods during the experiment (Table 1) show the transition from fast expansion to slow expansion as the leaf nears maturity. As seen in Figure 5 the relationship between the expansion rate and the observation day is approximately log-linear with an average correlation coefficient of 0.996 among the ten leaves studied. This suggests that the expansion rate of the leaves decreases exponentially during this phase. More frequent measurements could be used to gain a precise quantification of expansion rate changes over time.

### Mapping of leaf expansion

By colouring the marker-defined cells according to their expansion values we are able to produce maps showing the two-dimensional information of leaf expansion. The colour map, from blue, to red, indicates an increase in the rate of expansion.

**Figure 6:**
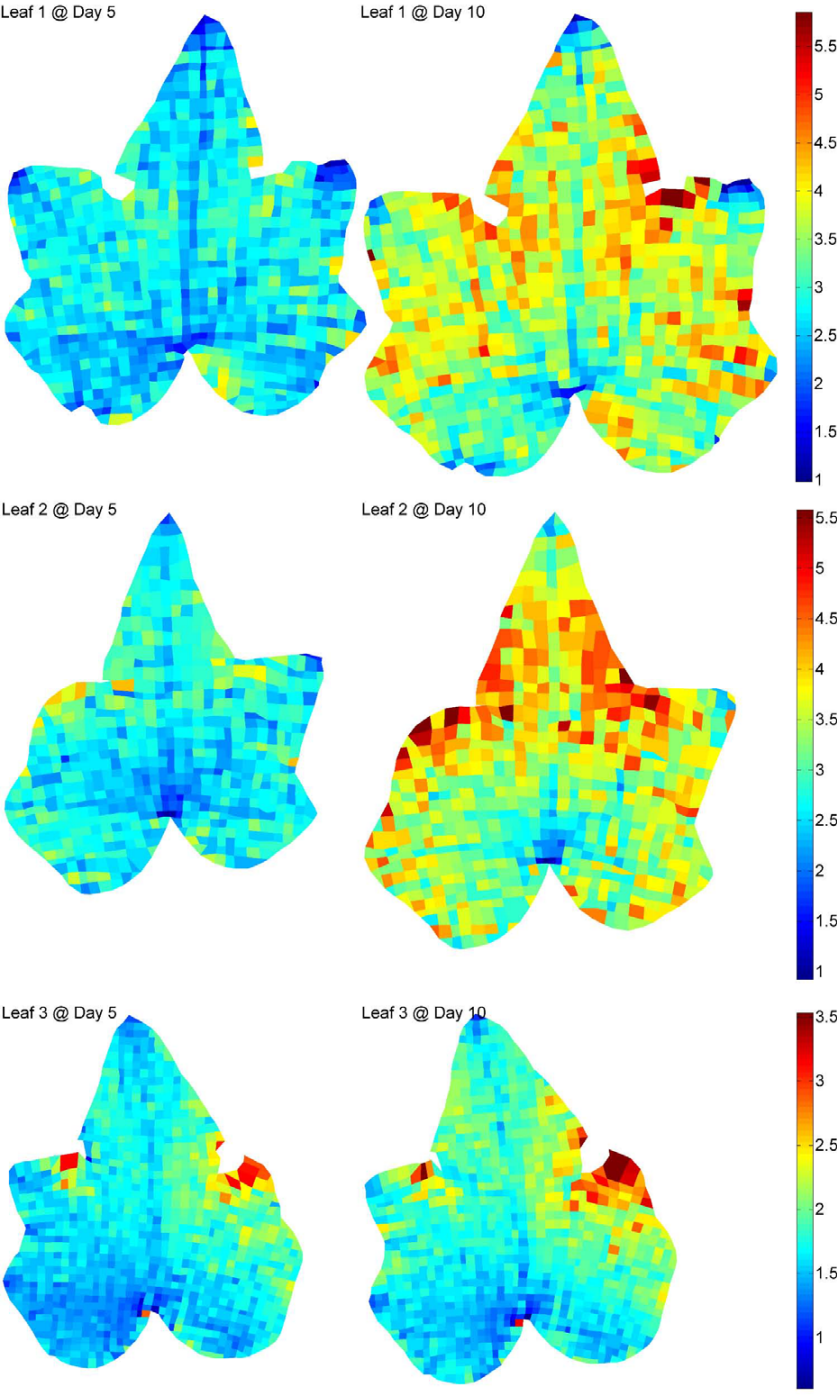
**Mapping of Leaves 1-3 in different growing stages**. The local expansion of the leaves is expressed through the color variation of each cell. The base of each leaf has the smallest degree of expansion and the sinus areas grow the fastest. Unlike Leaves 1 and 2, Leaf 3 shows a certain degree of asymmetry in its expansion.

Figure 6 shows the expansion maps for Leaves 1-3 at Day 5 and Day 10. The local expansion of the leaves is clearly expressed through the colour variation of each cell. Generally speaking, Leaves 1 and 2 have similar expansion rates throughout the entire experiment, both of which are larger than Leaf 3. In addition, the expansion of Leaves 1 and 2 is generally bilaterally symmetrical while Leaf 3 shows a certain degree of asymmetry in its expansion.

The non-uniform expansion of the leaves is clearly demonstrated by the expansion maps. We can see that the base of each leaf has the smallest degree of expansion, indicating this area consistently grows the slowest, called "cold spot". In contrast, the fastest expansion consistently occurs around the sinus areas between the terminal lobe and lateral lobes, called "hot spots", with an average expansion up to 3 times larger than the base area at Day 10. It is also clear from the results that the expansion rates along the primary veins, especially the midribs, are slower compared to their surrounding areas.

The expansion rate of each tip area is significantly correlated with the shape of that tip. A slower expansion rate will lead to a sharp tip, like most tips of the three leaves. For example, the tips of the terminal lobes show smaller expansion than their surrounding areas in all trials. In contrast, a faster expansion rate leads to a round-shaped tip.

To the best of our knowledge, spatial and temporal patterns of ivy leaf growth have not been reported before. Existing literature suggests that dicot leaves either grow with a base to tip growth gradient, or with a relatively uniform spatial growth [3]. In our results the sinus regions appear to have the highest rate of growth and the leaf base and major veins appear to have the slowest, which could be seen as a departure from the growth patterns of other studied dicot leaves. However, the gradual bending of the sinus regions of our ivy leaves over the experimental period is indicative of a higher rate of growth there, and partly validates the findings described above.

**Figure 7:**
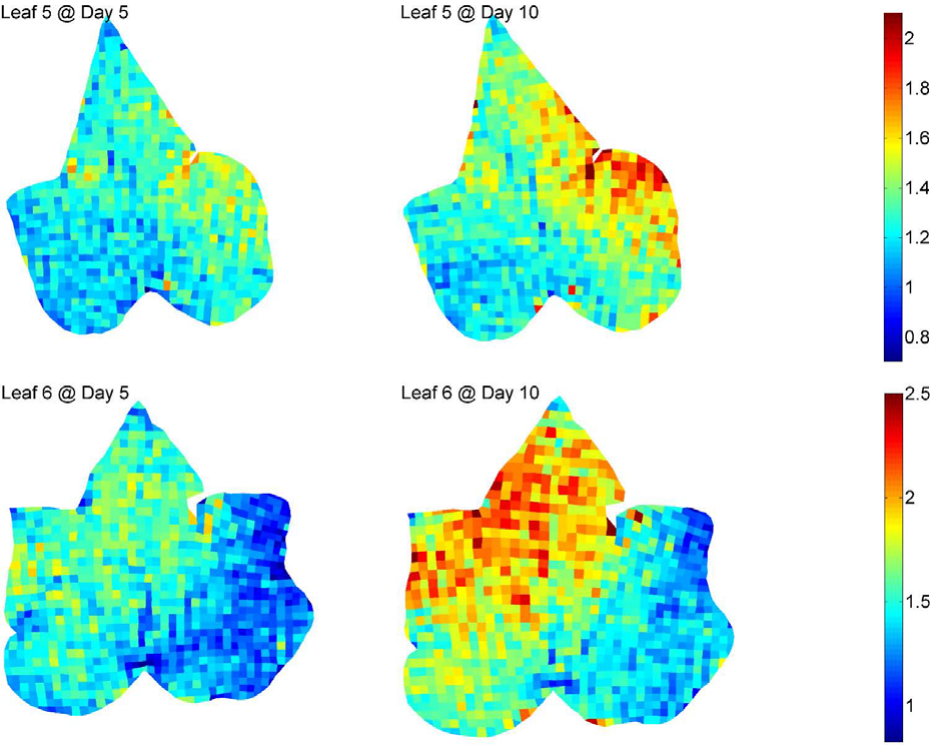
**Mapping of Leaves 5-6 in different growing stages**. Both leaves were partly covered using alumina foil, including the left side from the midrib of Leaf 5 and the right basal and lateral lobes of Leaf 6. The uncovered regions are expanding more quickly than the covered regions at both Day 5 and 10.

To further verify the validity and accuracy of our method, we intentionally covered part of two leaf surfaces (Leaves 5-6) with aluminium foil which would supposedly lead to asymmetric leaf expansion due to uneven illumination. Figure 7 shows the mapping results of both leaves at Days 5 and 10. It is very clear that the uncovered regions are expanding more quickly (mainly yellow and red) than the covered regions (blue) at both Day 5 and 10 leading to the absence of bilateral symmetry in the expansion patterns which commonly exists in normal leaves (Leaves 1-2).. Quantitatively, the overall expansion of the uncovered region is 1.4 and 1.6 times larger than the covered region at Day 10 in Leaves 5 and 6, respectively. Such a result is consistent with our hypothesis and in accordance with Sue et al., who characterized the expansion patterns of normal and malformed *Vitis vinifera *cv. *Ruby Red *leaves [14].

**Figure 8:**
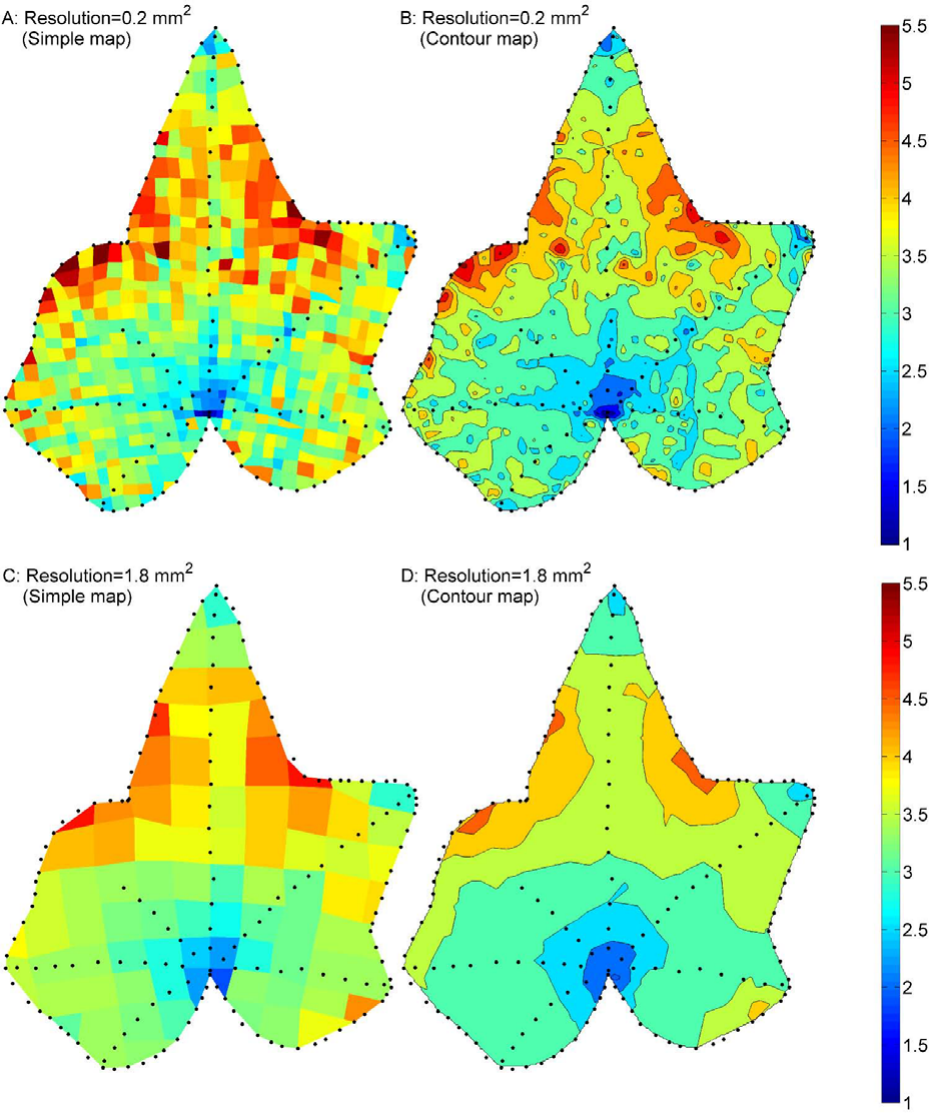
**Comparison of simple mapping and contour plotting with different printing resolutions**. Comparisons are made on Leaf 2 at Day 10. Black dots outlined the margin and major veins of the leaf. Contour plotting provides similar information to simple mapping with the expansion variation between regions more continuous. In lower-resolution maps, some important details are missing.

Contour plotting is another method to describe the expansion difference over an entire leaf surface. Figure 8(B) shows the colour-filled contour map of Leaf 2 at Day 10. Similar to the corresponding simple map (Figure 8A), it also shows the base to be the slowest expansion region and sinuses to be the fastest. The relationship between tip shapes and tip expansion rates are also demonstrated. The slower expansion along the veins (marked by black dots inside the map) becomes more visible in the contour map.

### Error analysis

As explained above, the 2D mapping of leaf expansion is comprised of three steps: pattern printing, optical recording and digital extraction. To study the accuracy and resolution of this technique, we performed error analysis on a patterned region containing 224 cells (including 190 square cells) for each of the three steps.

First, we determined the spatial resolution of the CCD camera and lens setup by finding the number of pixels per millimetre in an image with known dimensions. We found the resolution to be 86 pixels/mm, or 11.6 μm/pixel. This is thus the lower resolution limit for this method of expansion measurement given the camera used.

**Figure 9:**
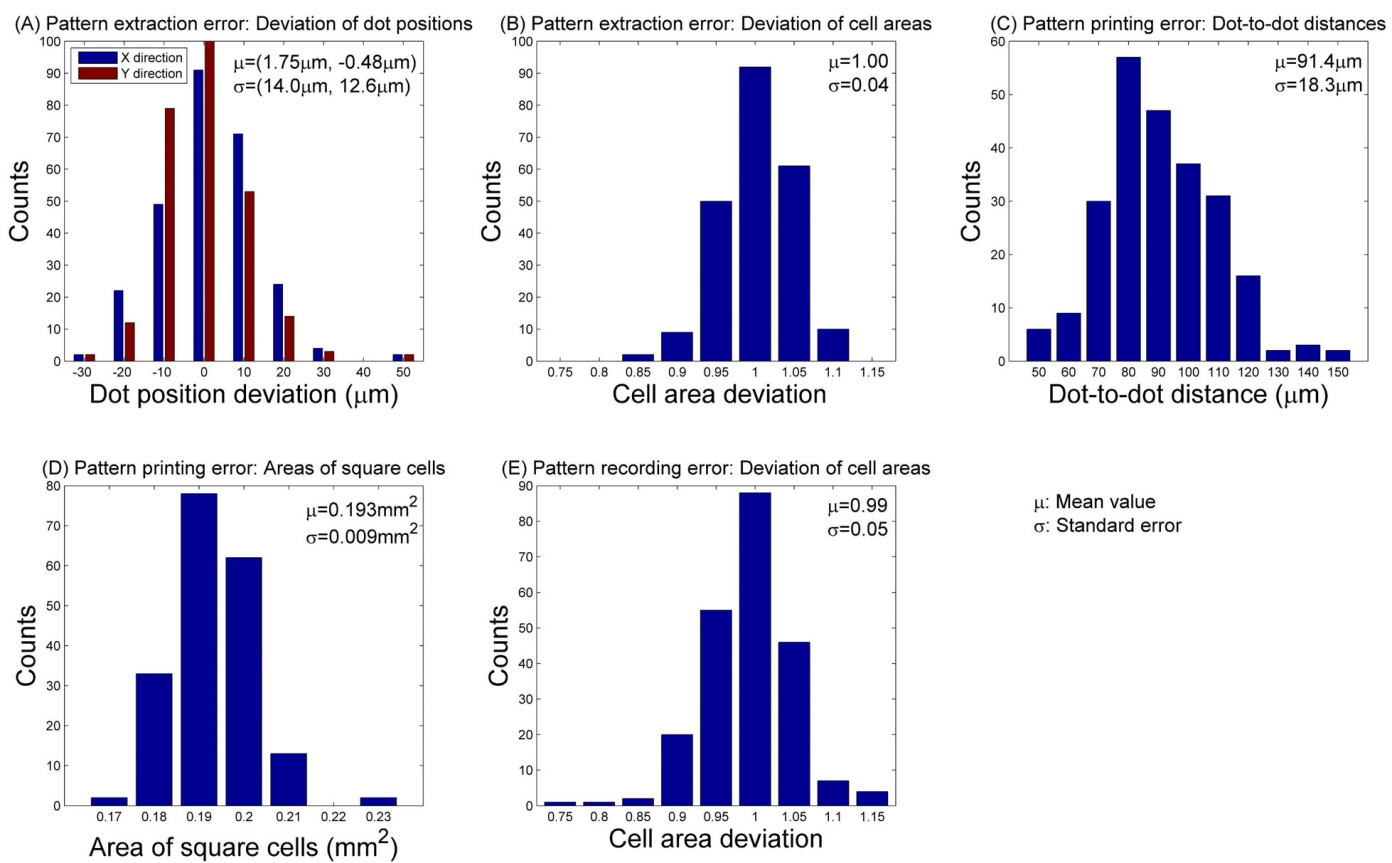
**Error analysis of pattern extraction, printing and recording processes**.

Because the digital pattern extraction process involves manually clicking on the image with a mouse, there is some degree of human error. To investigate this error we performed the extraction process twice on one image, resulting in two sets of 265 dot coordinates. First, the positional deviation between corresponding dots along the X and Y directions was calculated. Statistical results (Figure 9A) show that the error values (14.0 μm and 12.6 μm along the X and Y directions respectively) are larger than the spatial resolution of the images. Next we compared the areas of 224 cells from the two data sets and found a mean deviation of 1.00 and a standard error of 0.04 (Figure 9B). These results indicate that while the positional error is noticeable, the resulting error in cell area is negligibly small.

The accuracy of the physical printing process was determined in two steps. First, 240 pairs of adjacent printed dots were randomly chosen and their distances measured. The average distance was 91.4 μm with a standard error of 18.3 μm including the pattern extraction error. Figure 9C shows the distribution of the dot-to-dot distances. 91.4 μm is slightly larger than the expected dot distance of 87 μm, and 18.3 μm is substantially larger than the spatial resolution of the image. We suspect this error is due to inaccuracy in the movement of the stage, or the migration of the aqueous ink on the hydrophobic surface of the leaf. Although for this project the absolute positions of single dots are not important, precise study of surface properties and careful selection of inks would be necessary if more accurate printing is needed. The areas of a total of 190 square cells throughout the investigated region were also measured and their distribution analysed (Figure 9D). The average value is 0.19 mm^2 ^with a standard error of 0.01 mm^2^, which can be neglected.

Lastly there is some degree of error associated with imaging the leaves due to the physical action of setting up the camera and loading the sample. To measure this error we imaged a printed pattern twice, reloading the sample and reassembling the camera setup each time. We found the deviation in the areas of 224 cells to have a mean value of 0.99 and a standard error of 0.05 (Figure 9E). This error is the combination of both the pattern generation and recording processes. Through this analysis we have found that the total error is substantially smaller than both the overall leaf expansion (Table 1) and the expansion of individual cells (Figures 6 and 7), indicating that error does not have a significant impact on the results of this method.

## Discussion

Inkjet printing can deliver landmarks at fine scales and fast speeds. In the case of this research, by using 40 μm dots, a grid containing one thousand 0.2 mm^2 ^cells could be precisely printed in 10 min. Finer-scale printing can also be achieved using this method. This is a considerable improvement over the 25 mm^2 ^grids manually printed or stamped on leaves in classical growth studies and can potentially lead to the development of more sophisticated growth models encompassing a greater range of organs. In addition, because there is no contact between the nozzle and the organ surfaces in our custom built printer the plant organs are not mechanically damaged, as demonstrated by the control experiment described before.

To further study the influence of printing resolution on the measurements, we increased the grid cells from 0.2 mm^2 ^to 1.8 mm^2 ^in size by evenly selecting one out of three marker dots along both horizontal and vertical printed lines. The standard errors of deviation of cell areas are 0.01 during the pattern extraction process and 0.02 during the pattern recording process, both of which are substantially smaller than those of previously used finer grid (Figures 9B, 9E), indicating that a higher resolution would result in an increased measurement error. The mapping and contour plotting results are shown in Figure 8(C, D). Compared to the higher-resolution mapping results (Figure 8(A, B)), the "hot spots" and "cold spots" are also visible for expansion within one leaf, and the expansion variation from "hot spots" to "cold spots" becomes more continuous, virtually no isolated spots(Figure 8A, B). However, due to the reduced resolution some important details are missing. As an example, the slower expansion rate along the veins is not visible. In addition, the expansion disparity between "hot spots" and "cold spots" is underestimated. Since high-resolution printing provides additional detail while potentially increase measurement error, the choice of an appropriate resolution must be made according to the requirement of each study.

The main limitations of the system described here are leaf access and surface suitability. Young leaves are often protected by older leaves and leaf structures such as stipules. Maneuvering the shoot tip so that young leaves are accessible to the inkjet printer is a significant challenge. Our current printing system has a fixed head and moving stage and the machine as a whole cannot be tilted to access harder to reach parts of a plant. Climbing plants like ivy are therefore ideal as their shoots can be introduced at any orientation into the printing chamber. Future innovations should include redesign of the printer to provide adjustable stage orientation to give greater flexibility with regard to printable plant material. Many young leaves are covered with dense hairs and are rolled or folded into one of several patterns of space packing or "ptyxis" making these measurements challenging. With our present system evenly spaced landmarking can only be achieved if the leaf is relatively flat. We flatten leaves with gentle pressure, holding them in place using a weakly adhesive surface. This is often possible with leaves that are folded along the midrib (conduplicate). However, leaves that are tightly rolled (supervolute) when young are not treatable in this way and only the exposed surfaces can be landmarked. In addition, leaf flattening is also required during the pattern recording process, as in many other existing techniques. However, leaves of many species will experience significant distortion during expansion, leading to undulating surfaces which are difficult to be flattened and imaged.

In this study we used an opaque mark consisting of food colouring that provided sufficient contrast when transilluminated. However, the use of fluorescent markers would potentially be a way of gaining greater contrast for use with automated image analysis which would speed the process of tracking the landmarks.

We use non-contact inkjet printing here to place landmarks on leaves. However, the versatility of inkjet technology enables it to be used as a delivery system for a wide range of interesting components [15,16]. Examples relevant to plant science might include phytohormones delivered in precisely quantifiable amounts (perhaps along with markers) as quantitative bioassays. Besides dye, inkjet technology can be used as a particle delivery system. Increasingly, sensor technology is being reduced in scale [17,18] and small nanoparticles consisting of indicator chemicals can in theory be used to monitor physicochemical conditions on the leaf surface.

## Conclusions

In this article, we demonstrate the applicability of inkjet micropatterning to the study of plant growth and achieved high-resolution and two-dimensional monitoring of leaf expansion. Mapping results show that this method is able to describe the expansion difference at different regions of a leaf lamina on a very fine scale. The measurement is reliable according to error analysis and control experiments suggest that this technique can reflect the change of conditions during plant growth. As well as delivering landmarks, this technology could be used to deliver microscale targeted biological chemicals such as growth hormones, and possibly be used to pattern sensors directly on the leaves.

## Competing interests

The authors declare that they have no competing interests.

## Authors' contributions

LW raised the plant, developed the analysing program, carried out the expansion analysis and drafted the manuscript. STB built the inkjet printing system and carried out printing on the leaves. QCBC participated in the design of the study, provided support for the plant science aspects and helped draft the manuscript. KW conceived the study, participated in its design and coordination and helped draft the manuscript. All authors read and approved the final manuscript.
